# Patterns and Outcomes of Permanent Vascular Access in End-Stage Kidney Disease: A Multicenter Experience

**DOI:** 10.7759/cureus.94950

**Published:** 2025-10-19

**Authors:** Fatimah M Alhubail, Ali M Al Mousa, Ghadeer M Alhassan, Danah S Alali, Mona A Almutlaq, Mohammed Almulhim, Eliane El Tawil, Ghassan Salah, Amged Awad

**Affiliations:** 1 Internal Medicine, College of Medicine, King Faisal University, AlAhsa, SAU; 2 Nephrology, Almoosa Health Group, AlAhsa, SAU; 3 Nephrology, King Fahad General Hospital, AlAhsa, SAU; 4 Internal Medicine, College of Medicine, Al-Azhar University, Cairo, EGY

**Keywords:** arteriovenous fistula, arteriovenous graft, central venous catheter, end-stage kidney disease, hemodialysis, multicenter study, vascular access

## Abstract

Background

End-stage kidney disease (ESKD) is a growing health problem worldwide, with hemodialysis serving as the main treatment when transplantation is not feasible. Permanent vascular access is essential for effective dialysis, yet its patterns and outcomes remain variable across patient populations.

Aim

This multicenter study aimed to describe the patterns of vascular access use and evaluate the outcomes of permanent vascular access in patients with ESKD undergoing hemodialysis.

Methods

A retrospective multicenter cross-sectional study (January-June 2024) was conducted at two tertiary hospitals in Al-Ahsa, Saudi Arabia, including adults on maintenance hemodialysis via arteriovenous fistula (AVF), arteriovenous graft (AVG), or permanent catheter. Demographic, clinical, laboratory, and access data were extracted. Pending accesses were excluded from inferential analyses. Group comparisons used standard non-parametric and categorical tests; independent predictors of failure were estimated using logistic regression with two-sided α=0.05 (p<0.01 interpreted as strong evidence).

Results

Among 378 patients, permanent catheter use predominated [standard 193 (51.1%), long-standing 70 (18.5%)], followed by AVFs 113 (29.9%) and AVGs 2 (0.5%). Overall, 324 (85.7%) accesses were patent, 29 (7.7%) failed, and 25 (6.6%) were pending. In those with known status (n=353), access type was significantly associated with outcome: AVF failures 6 (5.3%), standard catheter failures 23 (13.7%), and no failures among long-standing catheters or AVGs-though these groups were small. Failed cases exhibited below-target hemoglobin levels. In multivariable analysis, hemoglobin below target (<12 g/dL) was independently associated with higher odds of failure (OR 0.17 for below vs within/above target, 95% CI 0.04-0.76; p=0.020), whereas older age (≥65 years) was associated with lower failure odds (OR 0.39, 95% CI 0.16-0.97; p=0.043).

Conclusions

In this multicenter experience, AVF use aligned with the most favorable patency profile. Anemia emerged as the most actionable correlate of failure, underscoring the importance of hemoglobin optimization around access creation and maintenance. Early planning for AVF and targeted surveillance, especially with anemia correction, was associated with greater permanent access durability.

## Introduction

End-stage kidney disease (ESKD), the terminal phase of chronic kidney disease (CKD), poses a profound global health challenge marked by irreversible renal failure requiring lifelong renal replacement therapy (RRT), predominantly hemodialysis (HD) [1]. With CKD affecting over 10% of the global population-over 800 million individuals-ESKD incidence continues to rise, driven by aging demographics, diabetes mellitus, hypertension, and obesity, particularly in low- and middle-income nations [1]. In Saudi Arabia, CKD prevalence is 5.7%, with high-risk groups showing suboptimal knowledge, attitudes, and practices (KAP) toward prevention and early detection [2]. This burden amplifies mortality risks, underscoring the need for robust vascular access (VA) strategies to sustain HD efficacy [1, 2].

Permanent VA is the lifeline for HD, yet it remains challenged by frequent complications, including maturation failure, stenosis, and dysfunction [3]. Guidelines endorse arteriovenous fistulas (AVFs) over arteriovenous grafts (AVGs) and central venous catheters (CVCs) because of their superior long-term patency and lower complication rates [3]. However, a substantial proportion of AVFs fail to mature due to factors such as intimal hyperplasia (IH), inadequate vascular remodeling, and CKD-related vasculopathy [4, 5]. Many patients, therefore, remain dependent on CVCs for prolonged periods, exposing them to higher risks of infection, thrombosis, and hospitalization [3, 4, 6, 7]. These persistent challenges reflect gaps in patient selection, access planning, and timely intervention, which together limit the effectiveness of current VA practices.

Several demographic, clinical, and biochemical factors have been implicated in VA dysfunction [8, 9]. Female sex, older age, obesity, peripheral and cerebrovascular disease, and small vein diameter (<2.5 mm) have all been associated with non-maturation [4-6, 10]. Laboratory abnormalities such as hyperphosphatemia, anemia, and thrombocytosis also compromise patency [8, 9]. Preoperative venous intimal hyperplasia has been linked to reduced blood flow and lower unassisted maturation, and prolonged use of temporary catheters increases failure risk [8, 11]. Targeted measures, including early planning, risk-based selection, and structured nursing interventions, have shown potential to improve outcomes [10], yet remain inconsistently implemented across settings [6,9].

Despite growing awareness of these risks, multicenter data describing real-world VA patterns and outcomes remain scarce, particularly in diverse ESKD populations where delays in planning often hinder timely AVF creation [2, 7]. This study seeks to address this gap by evaluating the distribution, predictors, and outcomes of permanent VA-AVFs, AVGs, and CVCs across multiple centers to inform strategies that promote earlier AVF use, reduce catheter dependence, and improve long-term hemodialysis outcomes.

## Materials and methods

Study design and setting

This retrospective multicenter cohort study was conducted between January 2024 and June 2024 to evaluate the patterns and outcomes of permanent vascular access in patients with end-stage kidney disease (ESKD). Data were obtained from two tertiary centers in Al-Ahsa, Saudi Arabia: Almoosa Specialist Hospital and Aljaber Kidney Center. The study was reported in accordance with the Strengthening the Reporting of Observational Studies in Epidemiology (STROBE) guidelines.

Eligibility criteria

Patients were eligible if they were 18 years of age or older, had a confirmed diagnosis of ESKD in accordance with Kidney Disease: Improving Global Outcomes (KDIGO) clinical practice criteria for chronic kidney disease, and were receiving maintenance hemodialysis through a permanent vascular access (arteriovenous fistula, arteriovenous graft, or permanent catheter). Patients were excluded if essential information on access type or site was missing, or if access status was recorded as pending at the time of evaluation. Other variables, such as body mass index or ejection fraction, were occasionally incomplete; these cases were retained in the study and coded as missing for analysis rather than excluded.

Data collection and variables

Demographic, clinical, and laboratory data were extracted manually from hospital records and entered into an electronic database for management and analysis. Collected variables included age, sex, smoking history, and anthropometric measurements, including height, weight, and body mass index (BMI). Clinical data included the primary etiology of ESKD and comorbidities such as hypertension, diabetes mellitus, ischemic heart disease, congestive heart failure, atrial fibrillation, sickle cell disease, cerebrovascular accidents, autosomal dominant polycystic kidney disease, hypothyroidism, benign prostatic hyperplasia, and other conditions that were grouped as “others” if observed in three or fewer patients.

Vascular access data included the type of access (arteriovenous fistula, arteriovenous graft, standard permanent catheter, or long-standing permanent catheter), the anatomical site of insertion, and access status (well-functioning, failed, or pending). Long-standing permanent catheter use was defined as continued dependence on permanent catheters due to medical or personal reasons, such as poor cardiac function, surgical contraindications, patient refusal of AVF/AVG, or recent diagnosis of ESKD. Reasons or barriers to establishing AVF/AVG were also collected, including surgical limitations, patient refusal, failed previous attempts, recent diagnosis of ESKD, or unknown causes.

Laboratory and clinical parameters

Laboratory and echocardiographic variables were categorized according to the cutoffs used by the participating dialysis centers, which reflect local practice standards for patients with CKD/ESKD and are based on treatment or monitoring targets rather than general population reference ranges. To ensure consistency, all continuous laboratory parameters were grouped into three categories representing dialysis-management thresholds: below target, within target, and above target. As several monitoring parameters contained missing data, values coded as “0” in the dataset were defined as missing in the statistics software to allow systematic exclusion from statistical tests without introducing analytical bias.

Hematologic and Nutritional Indices

Hemoglobin (g/dL) was categorized as below target (<12), within target (12-16), or above target (>16), consistent with anemia management practices. Albumin (g/L) was categorized as below target (<35), within target (35-50), or above target (>50). Ferritin (ng/mL) was categorized as below target (<500), within target (500-1000), or above target (>1000), reflecting dialysis-specific monitoring cutoffs routinely applied in anemia management within the participating centers. These thresholds correspond to the treatment and monitoring ranges used in real-world dialysis practice, focusing on iron availability rather than population-based reference values.

Metabolic and Biochemical Parameters

Creatinine (µmol/L) was categorized as below target (<53), within target (53-115), or above target (>115). Total cholesterol (mmol/L) was categorized as below target (<5.18) or above target (≥5.18), and triglycerides (mmol/L) as below target (<1.70) or above target (≥1.70).

Mineral and Bone Parameters

Calcium (mmol/L) was categorized as below target (<2.12), within target (2.12-2.62), or above target (>2.62). Phosphorus (mmol/L) was categorized as below target (<0.81), within target (0.81-1.45), or above target (>1.45). Parathyroid hormone (pg/mL) was categorized as below target (<15), within target (15-30), or above target (>30), reflecting CKD-mineral and bone disorder (MBD) monitoring thresholds used by the dialysis units.

Cardiac Function

Ejection fraction (%) was categorized as below target (<55), within target (55-70), or above target (>70). This standardized categorization facilitated coherent comparisons across biochemical and clinical parameters.

Statistical analysis

Data management and analysis were conducted using Microsoft Excel 2021 and SPSS Statistics version 26.0 (IBM Corp., Armonk, NY, USA). Continuous variables were first tested for normality using the Shapiro-Wilk test and inspection of histograms and Q-Q plots. As all variables violated normality assumptions, they were summarized as median with interquartile range (IQR) and compared between groups (patent vs failed access) using the Mann-Whitney U test with exact p-values. Categorical variables were expressed as counts and percentages, and group differences were assessed with the chi-square test or Fisher’s exact test when expected cell counts were <5. Logistic regression analysis was used to identify independent predictors of vascular access failure. A two-sided p-value <0.05 was considered statistically significant, with p <0.01 interpreted as strong evidence against the null hypothesis.

Ethical considerations

This study was approved by the Institutional Review Board of Almoosa Specialist Hospital (IRB Log No. ARC-24.1.03; NCBE Registration No. H-05-HS-100). Al-Jaber Kidney Center participated under the same multicenter protocol and ethical authorization. The requirement for informed consent was waived owing to the retrospective design and use of anonymized records. All procedures were conducted in accordance with the principles of the Declaration of Helsinki and local research governance regulations, ensuring strict protection of patient confidentiality and data privacy.

## Results

**Figure 1:**
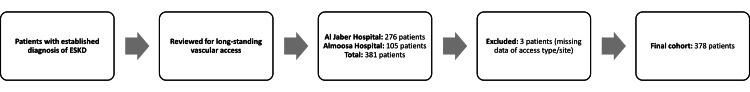
Flowchart of patient selection and inclusion

A total of 381 patients with established end-stage kidney disease (ESKD) were initially identified: 276 from Al Jaber Hospital and 105 from Almoosa Hospital. After excluding three patients due to missing essential information on vascular access type and site, 378 patients remained eligible for analysis (Figure 1).

The study population included 231 males (61.1%) and 147 females (38.9%). Smoking history was reported in six patients (1.6%), while 166 (43.9%) had an unreported smoking status. Regarding body mass index, 41 patients (10.8%) were underweight, 86 (22.8%) had normal weight, 81 (21.4%) were overweight, and 142 (37.6%) were obese. BMI classification could not be determined for 28 patients (7.4%) because either height or weight data were missing, as shown in Table 1.

**Table 1:** Baseline demographic and clinical characteristics of the study population (n=378) BMI: body mass index.

Characteristic		N (%)
Gender	Male	231 (61.1%)
	Female	147 (38.9%)
Smoking History	Smoker	6 (1.6%)
	Non-smoker	206 (54.5%)
	Unreported	166 (43.9%)
BMI	Underweight	41 (10.8%)
	Normal	86 (22.8%)
	Overweight	81 (21.4%)
	Obese	142 (37.6%)
	Unreported	28 (7.4%)

**Figure 2:**
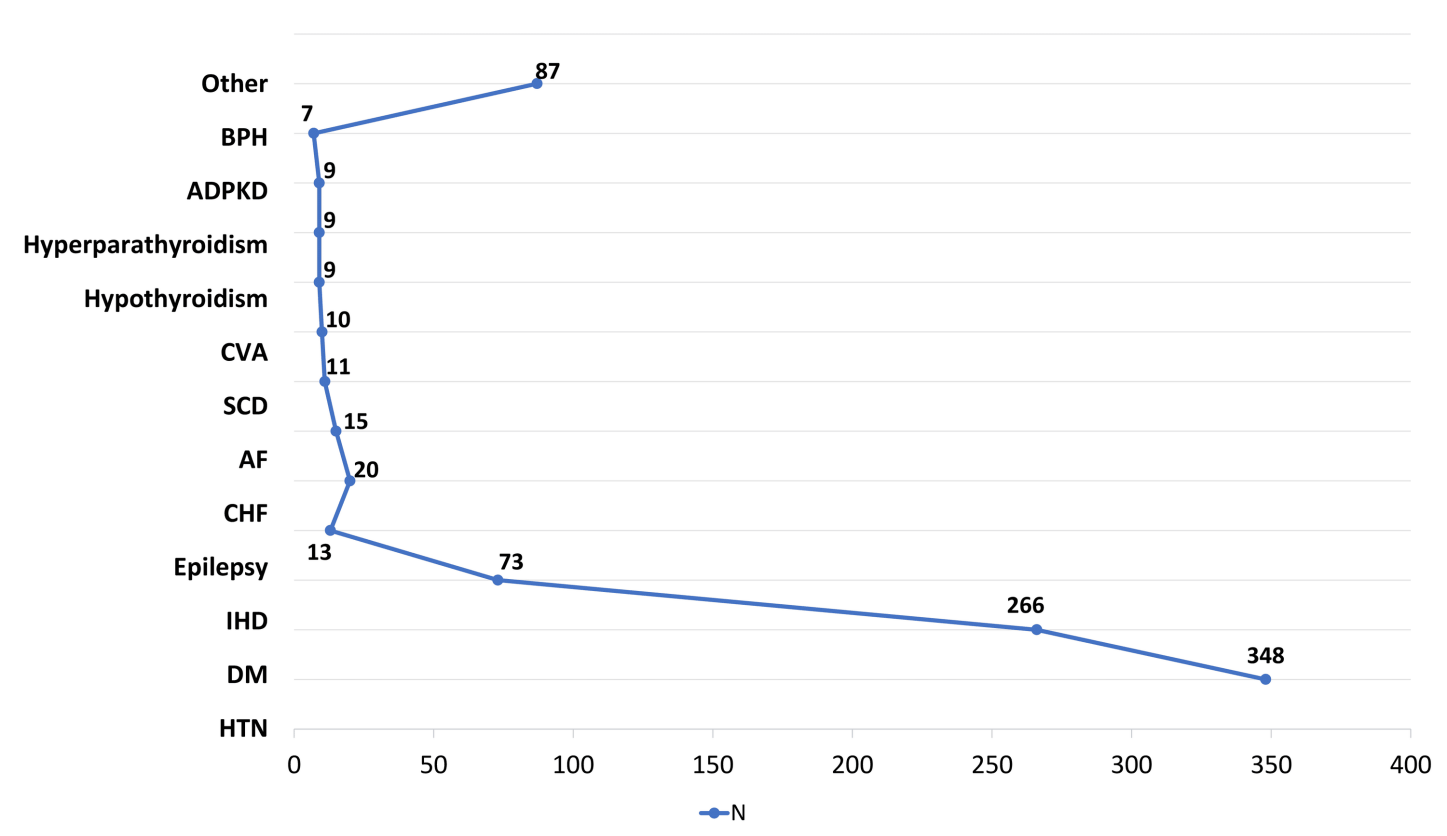
Distribution of Comorbidities Among Patients with End-Stage Kidney Disease (n=378) BPH: benign prostatic hyperplasia; ADPKD: autosomal dominant polycystic kidney disease; CVA: cerebrovascular accident; SCD: sickle cell disease; AF: atrial fibrillation; DM: diabetes mellitus; HTN: hypertension.

Hypertension was present in 348 patients (92.1%), diabetes mellitus in 266 (70.4%), and ischemic heart disease in 73 (19.3%). Epilepsy was documented in 13 patients (3.4%), congestive heart failure in 20 (5.3%), and atrial fibrillation in 15 (4.0%). Sickle cell disease was observed in 11 patients (2.9%), cerebrovascular accident in 10 (2.6%), and hyperparathyroidism in 9 (2.4%). Autosomal dominant polycystic kidney disease and hypothyroidism were each reported in 9 patients (2.4%), while benign prostatic hyperplasia was present in 7 patients (1.9%). The group of comorbidities classified as “others” encompassed a heterogeneous set of conditions, each occurring with very low frequency, and together accounted for 87 patients (23.0%). These included cardiovascular disorders (atrial flutter, valvular heart disease, ischemic cardiomyopathy, dilated cardiomyopathy, and cor pulmonale), hematologic and genetic conditions (glucose-6-phosphate dehydrogenase deficiency, Down syndrome, Alport syndrome, and renal agenesis), oncologic diseases (colon, uterine, breast, and thyroid malignancies, as well as multiple myeloma), neurological and psychiatric disorders (dementia, schizophrenia, depression, hydrocephalus, paraplegia, disc prolapse, and Alzheimer’s disease), metabolic and endocrine abnormalities (hyperthyroidism, parathyroid adenoma, adrenal adenoma, and hyperoxaluria), infectious diseases (hepatitis B, hepatitis C, human immunodeficiency virus, and tuberculosis sequelae), and other organ-specific disorders such as chronic lung disease (chronic obstructive pulmonary disease, bronchiectasis, and obstructive sleep apnea), hepatic cirrhosis, diverticulosis, nephrotic syndrome, lupus nephritis, solitary kidney, ureteric stricture, esophageal ulcer, pregnancy-related complications including pre-eclampsia, and vascular complications such as steal syndrome. The full distribution of comorbidities is presented in Figure 2.

Among arteriovenous fistulas, the brachio-cephalic site was the most common, observed in 62 patients (54.9%), followed by radio-cephalic in 45 (39.8%), brachio-basilar in 5 (4.4%), and brachio-axillary in 1 (0.9%). For standard permanent catheters, the internal jugular vein was used in 183 patients (94.8%) and the femoral vein in 10 (5.2%). Long-standing permanent catheters were placed in 66 patients (94.3%) via the internal jugular vein and in 4 (5.7%) via the femoral vein. Two patients (100.0%) had an arteriovenous graft placed at the radio-cephalic site (Table 2).

**Table 2:** Distribution of vascular access types and sites among the study population * Subset of patients remained on permanent catheters long-term due to medical or personal reasons e.g., poor cardiac function, surgical barriers, refusal of arteriovenous fistula (AVF)/ arteriovenous graft (AVG), or recent diagnosis of end-stage kidney disease (ESKD).

Access Type		Access Site	N (%)	Total N (%)
Arteriovenous Fistula		Brachio-axillary	1 (0.9%)	113 (29.9%)
		Brachio-basilar	5 (4.4%)	
		Brachio-cephalic	62 (54.9%)	
		Radio-cephalic	45 (39.8%)	
Permanent Catheter	Standard	Femoral	10 (5.2%)	193 (51.1%)
		Internal jugular	183 (94.8%)	
	Long-standing*	Femoral	4 (5.7%)	70 (18.5%)
		Internal jugular	66 (94.3%)	
Arteriovenous Graft		Radio-cephalic	2 (100.0%)	2 (0.5%)

A total of 324 vascular accesses (85.7%) were patent, 29 (7.7%) were classified as failed, referring to accesses that had lost functionality and were no longer usable for dialysis, whether due to complications such as thrombosis or stenosis or because they required revision with a graft, and 25 (6.6%) were pending, representing patients whose permanent access had not yet been established. The overall distribution of vascular access status is shown in Table 3.

**Table 3:** Vascular access patency status in the study population (n=378)

Status	N (%)
Patent access	324 (85.7%)
Failed access	29 (7.7%)
Pending access	25 (6.6%)
Total	378 (100.0%)

**Figure 3:**
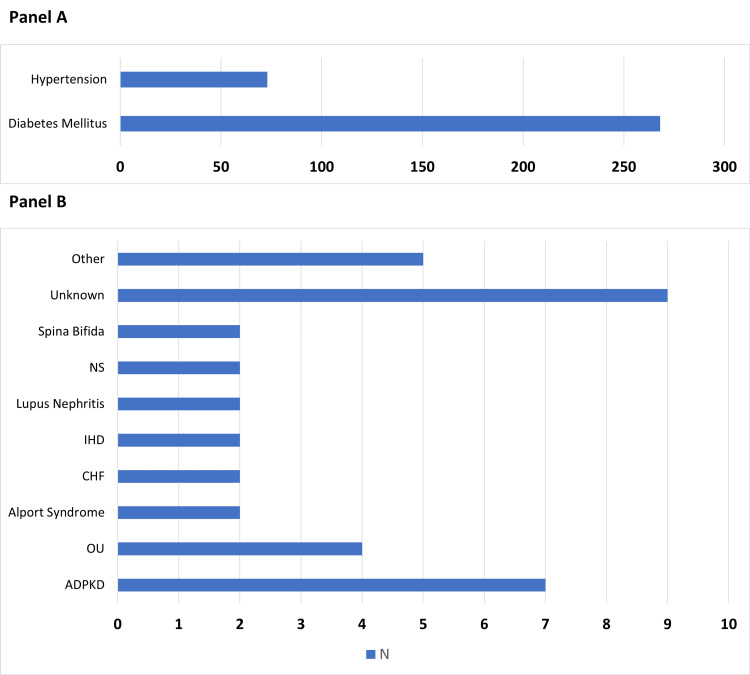
Primary causes of end-stage kidney disease among the study population (n=378) Panel A shows the most frequent causes (diabetes mellitus and hypertension). Panel B presents the less frequent causes, grouped for clarity. ADPKD: autosomal dominant polycystic kidney disease; OU: obstructive uropathy; IHD: ischemic heart disease; CHF: congestive heart failure; NS: nephrotic syndrome.

The most frequent primary cause of ESKD in the study population was diabetes mellitus, observed in 268 patients (70.9%), followed by hypertension in 73 patients (19.3%). Less common etiologies included autosomal dominant polycystic kidney disease in 7 patients (1.9%) and obstructive uropathy in 4 patients (1.1%). Rare causes, each affecting 2 patients (0.5%), comprised Alport syndrome, congestive heart failure, ischemic heart disease, lupus nephritis, nephrotic syndrome, and spina bifida. The etiology was unknown in 9 patients (2.4%), while other uncommon conditions were grouped together and accounted for 5 patients (1.3%). The full distribution of primary causes is presented in Figure 3, with Panel A highlighting the most frequent etiologies and Panel B displaying the less common causes.

Laboratory parameters of the study population are summarized in Table 4. Hemoglobin was below target in 270 patients (71.4%), within target in 107 (28.3%), and above target in 1 (0.3%). Albumin was below target in 129 patients (34.3%), within target in 246 (65.4%), and above target in 1 (0.3%). Creatinine was above target in 364 patients (96.6%), within target in 10 (2.7%), and below target in 3 (0.8%). Most patients had within-target cholesterol levels (329, 89.2%), while 40 (10.8%) had above target values. Triglycerides were within target in 251 patients (69.0%) and above target in 113 (31.0%). Calcium was below target in 197 patients (52.5%), within target in 177 (47.2%), and above target in 1 (0.3%). Phosphorus was above target in 222 patients (59.0%), within target in 128 (34.0%), and below target in 26 (6.9%). Parathyroid hormone was above target in 266 patients (72.1%), within target in 64 (17.3%), and below target in 39 (10.6%). Ferritin was below target in 195 patients (51.9%), within target in 100 (26.6%), and above target in 81 (21.5%). Ejection fraction values were available for 261 patients, of whom 145 (55.6%) were within target and 116 (44.4%) were below target; no patient was classified as above target. Missing values were present across several parameters and were handled consistently in the analysis.

**Table 4:** Laboratory parameters of the study population “–” indicates categories that are not applicable, while “0 (0.0%)” denotes applicable categories with no observed cases. Target thresholds were defined according to dialysis-center monitoring standards as described in the methods.

Parameter	Total N	Below target N (%)	Within target N (%)	Above target N (%)	Mean ± SD
Hemoglobin (g/dL)	378	270 (71.4%)	107 (28.3%)	1 (0.3%)	10.9 ± 2.0 g/dL
Albumin (g/L)	376	129 (34.3%)	246 (65.4%)	1 (0.3%)	36.0 ± 5.0 g/L
Creatinine (µmol/L)	377	3 (0.8%)	10 (2.7%)	364 (96.6%)	428.7 ± 268.1 µmol/L
Cholesterol (mmol/L)	369	-	329 (89.2%)	40 (10.8%)	4.45 ± 12.97 mmol/L
Triglycerides (mmol/L)	364	-	251 (69.0%)	113 (31.0%)	1.48 ± 0.89 mmol/L
Calcium (mmol/L)	375	197 (52.5%)	177 (47.2%)	1 (0.3%)	2.12 ± 0.22 mmol/L
Phosphorus (mmol/L)	376	26 (6.9%)	128 (34.0%)	222 (59.0%)	1.61 ± 0.56 mmol/L
Parathyroid hormone (pg/mL)	369	39 (10.6%)	64 (17.3%)	266 (72.1%)	71.0 ± 65.6 pg/mL
Ferritin (ng/mL)	375	195 (51.9%)	100 (26.6%)	81 (21.5%)	859.8 ± 2315.5 ng/mL
Ejection fraction (%)	261	116 (44.4%)	145 (55.6%)	0 (0.0%)	50.0 ± 10.1 %

Barriers to establishing arteriovenous access were documented in 113 cases (Table 5). Among individuals with standard permanent catheters, the most frequent reasons were recent diagnosis of ESKD in 26 subjects (63.4%) and failed previous attempts in 15 cases (36.6%). In contrast, long-standing permanent catheter use was most often attributed to surgical contraindications in 29 individuals (41.4%), followed by unknown causes in 26 cases (37.1%) and refusal in 15 subjects (21.4%). These findings indicate that while standard catheters were generally used as a temporary solution for newly diagnosed cases or following access failure, long-standing catheters reflected persistent medical or personal barriers that prevented the creation of AVF/AVG.

**Table 5:** Reported reasons or barriers to establishing arteriovenous access (AVF/AVG) PC: permanent catheter.

Reason	Standard PC N (%)	Long-standing PC N (%)	Total N (%)
Surgical	0 (0.0%)	29 (41.4%)	29 (25.7%)
Refusal	0 (0.0%)	15 (21.4%)	15 (13.3%)
Unknown	0 (0.0%)	26 (37.1%)	26 (23.0%)
Failed	15 (36.6%)	0 (0.0%)	15 (13.3%)
Recently diagnosed	26 (63.4%)	0 (0.0%)	26 (23.0%)
Total	41 (100.0%)	70 (100.0%)	113 (100.0%)

Episodes of vascular access failure or non-maturation were identified in 94 patients, corresponding to 100 recorded events, because some individuals experienced more than one complication (Table 6). The most frequent causes were thrombosis, present in 41 cases (43.6%), and stenosis in 37 cases (39.4%), followed by non-maturation in 13 cases (13.8%) and unknown causes in 16 (17.0%). Less common events included venous hypertension (7, 7.4%) and aneurysm (3, 3.2%). Other isolated complications, each affecting ≤2 cases (7 events in total), comprised AVF aneurysmal dilation, hematoma, pseudo-aneurysm, seroma, Candida infection, chronic hypotension, and repeated angioplasties.

Statistical testing demonstrated that thrombosis was significantly associated with a higher number of recurrent episodes, with its proportion increasing from 28.6% among single-episode cases to 60.7% in those with two episodes and 66.7% in those with three or more (χ²=10.97, p=0.004). Chronic hypotension also reached statistical significance, being observed only in individuals with three or more episodes (χ²=10.21, p=0.006), although this finding was based on a single case and should be interpreted with caution.

**Table 6:** Episodes of vascular access failure and their recorded causes (N=94) ** p<0.01; ^Fisher’s exact test applied when expected cell count <5. Row totals exceed the number of affected cases (N=94) because some individuals experienced multiple complications. Column totals represent mutually exclusive frequency categories and sum to 100 events.

Cause of failure / non-maturation	Once N (%)	Twice N (%)	≥3 N (%)	Total N (%)	χ² value	p-value
Thrombosis	18 (28.6)	17 (60.7)	6 (66.7)	41 (43.6)	10.97	0.004**
Stenosis	20 (31.7)	13 (46.4)	4 (44.4)	37 (39.4)	2.03	0.363
Non-maturation	7 (11.1)	4 (14.3)	2 (22.2)	13 (13.8)	0.92	0.632^
Unknown	12 (19.0)	2 (7.1)	2 (22.2)	16 (17.0)	2.33	0.312^
Venous hypertension	4 (6.3)	2 (7.1)	1 (11.1)	7 (7.4)	0.28	0.871^
Aneurysm	1 (1.6)	1 (3.6)	1 (11.1)	3 (3.2)	2.50	0.287^
Chronic hypotension	0 (0.0%)	0 (0.0%)	1 (11.1)	1 (1.1)	10.21	0.006**^
Other rare causes	6 (9.5)	1 (3.6)	0 (0.0%)	7 (7.4)	0 (0.0%)	0 (0.0%)
Total (column)	63 (67.0)	28 (29.8)	9 (9.6)	100 (100)	-	-

Among 353 individuals, Table 7 demonstrates the associations between demographic, clinical, laboratory, and access-related factors and vascular access outcomes (patent vs. failed; pending cases excluded). Access type showed the strongest evidence of association with outcome. AVFs achieved the highest patency (94.7%) with a failure rate of only 5.3%, while standard permanent catheters exhibited the greatest failure proportion at 13.7%. Long-standing catheters and grafts recorded no failures in this cohort, though interpretation is constrained by the limited numbers in these categories (n=70 and n=2, respectively). The chi-square statistic provided strong evidence for a relationship between access type and outcome (χ²=14.39, p=0.001), clearly favoring AVFs.

**Table 7:** Factors associated with vascular access patency (patent vs failed) among individuals with known status (n=353) **p<0.01; ^Fisher’s exact test applied when expected counts <5; “–” indicates categories that are not applicable. AVF: arteriovenous fistula; AVG: arteriovenous graft; PC: permanent catheter.

Variable	Level	Patent N (%) / Median [IQR]	Failed N (%) / Median [IQR]	Test statistic	p-value
Gender	Male	191 (89.7%)	22 (10.3%)	χ²=3.18	0.079^
	Female	133 (95.0%)	7 (5.0%)		
Smoking history	Smoker	3 (60.0%)	2 (40.0%)	χ²=7.55	0.051^
	Non-smoker	183 (93.4%)	13 (6.6%)		
	Unreported	138 (90.8%)	14 (9.2%)		
Age (years)	-	59 [25.0]	65 [24.0]	U=3811.5	0.092
Access type	AVF	107 (94.7%)	6 (5.3%)	χ²=14.39	0.001**^
	Standard PC	145 (86.3%)	23 (13.7%)		
	AVG	2 (100.0%)	0 (0.0%)		
	Long-standing PC	70 (100.0%)	0 (0.0%)		
Hemoglobin (g/dL)	-	11.0 [2.5]	10.4 [2.0]	U=3303	0.008**
Albumin (g/L)	-	36.1 [5.2]	35.9 [3.4]	U=3662.5	0.054
Creatinine (µmol/L)	-	382 [368.0]	608 [520.0]	U=4140.5	0.301

Smoking history yielded borderline evidence of association (p=0.051), with 40.0% of smokers experiencing access failure compared with 6.6% of non-smokers. Gender also approached significance (p=0.079), with higher failure observed in males (10.3%) relative to females (5.0%). Median age was higher among failed cases (65 years) compared to patent ones (59 years), a difference that trended toward association (p=0.092).

Among laboratory parameters, lower hemoglobin demonstrated strong evidence of association with failure (median 10.4 g/dL vs. 11.0 g/dL; Mann-Whitney U=3303, p=0.008). Albumin levels were lower in failed cases, though only borderline evidence was observed (p=0.054). Creatinine was higher in the failed group (608 vs. 382 µmol/L), but without supporting evidence of association (p=0.301). Other biochemical parameters, including cholesterol, triglycerides, calcium, phosphorus, parathyroid hormone, ferritin, and ejection fraction, showed no evidence of association with outcome.

Additional variables were also investigated, including BMI classification, hypertension, diabetes, ischemic heart disease, epilepsy, congestive heart failure, atrial fibrillation, sickle cell disease, cerebrovascular accident, hyperparathyroidism, autosomal dominant polycystic kidney disease, hypothyroidism, benign prostatic hyperplasia, morbid obesity, grouped ‘other’ comorbidities, and access site; however, none of these demonstrated evidence of association with access outcome in this cohort (all p≥0.12).

In the multivariable logistic regression model (Table 8), two factors emerged as independent predictors of vascular access patency status. Low hemoglobin (<12 g/dL) was strongly associated with higher odds of access failure (OR 0.17, 95% CI 0.04-0.76, p=0.020). This finding underscores the clinical importance of anemia management in dialysis patients, as it appears to compromise vascular access outcomes. Older age (≥65 years) was paradoxically protective, being associated with lower odds of failure (OR 0.39, 95% CI 0.16-0.97, p=0.043). This counterintuitive effect may reflect survival bias, where elderly patients with longstanding functional access are over-represented in the cohort.

**Table 8:** Multivariable logistic regression analysis of predictors of vascular access failure (n=353) * p<0.05; OR: odds ratio; CI: confidence interval; BMI: body mass index; PTH: parathyroid hormone; EF: ejection fraction.

Predictor	OR (95% CI)	p-value
Obesity: BMI ≥30 kg/m²	1.39 (0.56 – 3.45)	0.476
Smoker	0.37 (0.04 – 3.39)	0.379
Male sex	0.38 (0.15 – 1.00)	0.050
Age ≥65 years	0.39 (0.16 – 0.97)	0.043*
Parathyroid hormone >30 pg/mL	2.07 (0.48 – 8.92)	0.328
Ferritin >1000 ng/mL	2.21 (0.91 – 5.38)	0.081
Ejection fraction <55%	1.15 (0.45 – 2.92)	0.774
Phosphorus >1.45 mmol/L	0.87 (0.37 – 2.02)	0.738
Calcium <2.12 mmol/L or >2.62 mmol/L	1.06 (0.46 – 2.42)	0.898
Triglycerides ≥1.70 mmol/L	1.10 (0.43 – 2.84)	0.846
Albumin <35 g/L	0.76 (0.31 – 1.86)	0.553
Cholesterol ≥5.18 mmol/L	2.00 (0.42 – 9.56)	0.385
Hemoglobin <12 g/dL	0.17 (0.04 – 0.76)	0.020*

Male sex showed a trend toward reduced failure risk (OR 0.38, p=0.050), narrowly missing statistical significance, suggesting possible sex-related biological or anatomical influences on access durability. Ferritin levels >1000 ng/mL demonstrated a borderline association with higher failure risk (OR 2.21, p=0.081), hinting at potential vascular damage related to iron overload. Other variables, including obesity, smoking, biochemical derangements (albumin, calcium, phosphorus, triglycerides, cholesterol), left ventricular ejection fraction, and parathyroid hormone, were not independently predictive after adjustment.

The overall model demonstrated acceptable fit (Hosmer-Lemeshow p=0.493) and explained approximately 16% of the variance in access failure (Nagelkerke R²=0.163). These results indicate that while several laboratory and clinical parameters contribute to patency in univariate analysis, anemia remains the most robust independent predictor in this population.

## Discussion

Providing care for patients with ESKD is often challenging, with much of the difficulty centered on securing reliable vascular access that can sustain long-term treatment and preserve quality of life. In our cohort, AVFs demonstrated the most favorable long-term patency, with 107 (94.7%) patent and 6 (5.3%) failed, whereas standard permanent catheters showed 145 (86.3%) patent and 23 (13.7%) failed, reinforcing their role as the gold standard for hemodialysis access. Previous studies have also highlighted their advantages, including lower risks of infection and thrombosis as well as reduced healthcare costs [12-15]. According to previous literature, permanent catheters have been identified as independent predictors of mortality in hospitalized patients undergoing hemodialysis [16]. The continued reliance on central venous catheters in a substantial proportion of patients is concerning, given their markedly inferior outcomes compared with arteriovenous fistulas. In a nationwide cohort of 183,490 hemodialysis patients, the adjusted hazard ratios for all-cause mortality were 1.30 for arteriovenous grafts, 1.56 for arterial superficialization, and 2.15 for tunneled cuffed central venous catheters, compared with arteriovenous fistula (AVF) as the reference. For infection-related mortality, tunneled catheters showed the highest risk with an adjusted hazard ratio of 2.75, while AVFs were associated with the lowest risk [17]. Similarly, another study emphasized that central venous catheter (CVC) use carries higher rates of infection and thrombosis and is less safe than AVFs, which offer greater longevity and lower complication rates [9].

Moreover, many patients faced barriers to arteriovenous fistula (AVF) creation, most commonly surgical contraindications 29 (25.7%), recent diagnosis of ESKD 26 (23.0%), which may reflect delayed referral, unknown causes 26 (23.0%), and patient refusal 15 (13.3%) (Table 5). These findings are consistent with previous reports showing that delayed referral, lack of pre-dialysis vascular mapping, and social or systemic barriers limiting timely surgical access are major contributors to the continued use of central venous catheter (CVC) at dialysis initiation [18].

An additional and more unexpected finding was the role of hemoglobin <12 g/dL. Patients with hemoglobin <12 g/dL were more likely to develop access failure, often with recurrent episodes. This was consistent with the study by Yap et al., where hemoglobin <12 g/dL was associated with early failure in DM patients [19]. Anemia is a frequent and clinically significant complication of CKD, arising from multiple factors such as reduced erythropoietin production, iron-restricted erythropoiesis, persistent inflammation, and tissue hypoxia [20]. This relationship may not be coincidental; anemia in ESKD can lead to a vicious cycle of impaired tissue oxygenation, chronic inflammation, and vascular changes that could predispose to clot formation [21]. While anemia management is already a core element of dialysis care [22], our data suggest it may also have implications for maintaining vascular access. This connection has not been emphasized strongly in previous reports.

Previous studies, including that by Montagnana et al., have shown that patients on chronic hemodialysis are particularly vulnerable to thrombotic complications, such as arterial events and vascular access thrombosis [23]. In line with these findings, our analysis demonstrated a significant association between thrombosis and recurrent access failure. The pathogenesis of access failure is largely driven by impaired vascular remodeling and neointimal hyperplasia, which predispose to stenosis and thrombosis. Systemic therapies with pleiotropic effects, including antiplatelets, omega-3 fatty acids, statins, and renin-angiotensin-aldosterone system (RAAS) inhibitors, may help mitigate these complications by enhancing access maturation and exerting anti-inflammatory and vaso-protective effects, making them potential adjunctive strategies for ESKD patients on dialysis [24].

In our study, patients aged ≥65 years showed a lower risk of access failure, aligning with findings from previous reports [25-27]. This association may reflect survival bias rather than genuine biological protection. Variations in reported AVF patency rates among elderly populations may stem from differences in age classifications, underlying comorbidities, and local clinical practice patterns [9]. Consistently, evidence from a large meta-analysis indicates that older age alone does not adversely affect the long-term patency of autogenous accesses like arteriovenous fistulas, suggesting that age should not be considered a barrier to their use in appropriately selected patients [28]. However, age-related physiological changes, including vascular calcification, endothelial dysfunction, and reduced vascular remodeling capacity, would ordinarily be expected to impair access maturation and durability, making the observed protective effect in this cohort attributable to the selective survival of healthier elderly individuals likely possible.

Other laboratory and clinical parameters, including calcium <2.12 mmol/L or >2.62 mmol/L, phosphorus >1.45 mmol/L, ferritin >1000 ng/mL, triglycerides ≥1.70 mmol/L, cholesterol ≥5.18 mmol/L, albumin <35 g/L, and ejection fraction <55%, were not independently associated with outcome, which contrasted with similar studies, where AVF dysfunction is highly associated with several risk factors, which included weight, phosphorus >1.45 mmol/L, and sex [9, 28-30]. Comorbidities such as diabetes and cardiovascular disease have been associated with shorter patency life compared to non-diseased patients [31]. While other studies showed that AVF patency didn’t differ between diabetics and non-diabetics [32, 33]. The lack of association in our cohort may reflect differences in patient selection, local dialysis practices, or sample size, highlighting the need for larger, multicenter studies to clarify these relationships and identify context-specific risk factors for access failure.

Our study indicated that male sex showed a trend toward reduced access failure, narrowly missing statistical significance. This aligns with Bashar et al., who reported that AVF non-maturation was more commonly observed in women [34]. While crude failure proportions were higher in males (10.3%) than in females (5.0%), this difference was not statistically significant (p=0.079). In the adjusted model, male sex was not an independent predictor, though it showed a non-significant trend toward reduced failure risk (p=0.050), suggesting that the unadjusted difference likely reflects variation in access type distribution rather than a true biological effect. Several studies have demonstrated that women tend to have lower AVF maturation rates and longer maturation times compared to men, likely due to smaller average vessel diameters, reduced vasodilation capacity, and limited outward remodeling potential [4, 30, 35-37].

To the best of the authors’ knowledge, this is the first study to highlight patterns and outcomes of access failure among hemodialysis patients in AlAhsa, Saudi Arabia. Moreover, this study population was drawn from two major governmental dialysis centers, which ensures a relatively homogenous cohort. A multivariate logistic regression analysis was also implemented to adjust for potential confounders and increase the credibility of this study’s findings. Additionally, the inclusion of comprehensive demographic, clinical, and laboratory data enabled the identification of actionable predictors of access failure, such as hemoglobin <12 g/dL, providing clinically relevant insights that may inform anemia management and access planning.

Although this study had several strengths, it was not free of limitations. This multifactorial analysis underscores the complex interplay between systemic and localized factors that influence vascular access outcomes. However, one key limitation is that the analysis was cross-sectional and based on retrospective records, precluding time-to-event assessment or inference about temporal relationships between predictors and outcomes. Several important procedural parameters, such as vein diameter, surgical technique, and vessel histology, were not captured in the dataset. Furthermore, the retrospective design is inherently subject to biases related to incomplete or inaccurate documentation in medical records. Manual data extraction, while necessary, may have introduced minor errors or misclassifications of variables, potentially affecting data reliability. Finally, the study’s geographic confinement to two centers in a single region may limit the generalizability of these findings to other populations with differing demographic or healthcare characteristics.

## Conclusions

In this multicenter study of 378 patients with end-stage kidney disease, vascular access outcomes differed substantially by access type and patient characteristics. Arteriovenous fistulas demonstrated the most favorable patency, with 6 (5.3%) failures, whereas standard permanent catheters failed in 23 (13.7%) of cases. In adjusted analysis, hemoglobin <12 g/dL independently predicted access failure (OR 0.17, 95% CI 0.04-0.76; p=0.020), underscoring the importance of anemia management in maintaining access function. Age ≥65 years was associated with reduced failure risk (OR 0.39, 95% CI 0.16-0.97; p=0.043), a finding that may reflect survival bias rather than true biological protection. Other laboratory and clinical parameters, including calcium <2.12 mmol/L or >2.62 mmol/L, phosphorus >1.45 mmol/L, ferritin >1000 ng/mL, triglycerides ≥1.70 mmol/L, cholesterol ≥5.18 mmol/L, albumin <35 g/L, and ejection fraction <55%, were not independently associated with outcome.

These findings emphasize the clinical priority of early AVF creation and anemia control to optimize long-term dialysis access. Future prospective studies with larger cohorts and time-to-event designs are required to validate these associations and clarify the biological mechanisms underlying vascular access failure.
